# A brief questionnaire for measuring alarm fatigue in nurses and physicians in intensive care units

**DOI:** 10.1038/s41598-023-40290-7

**Published:** 2023-08-24

**Authors:** Maximilian Markus Wunderlich, Sandro Amende-Wolf, Henning Krampe, Jochen Kruppa, Claudia Spies, Björn Weiß, Belinda Memmert, Felix Balzer, Akira-Sebastian Poncette

**Affiliations:** 1 Institute of Medical Informatics, Charité – Universitätsmedizin Berlin, Corporate Member of Freie Universität Berlin and Humboldt-Universität zu Berlin, Berlin, Germany; 2 Department of Anesthesiology and Intensive Care Medicine, Charité – Universitätsmedizin Berlin, Corporate Member of Freie Universität Berlin, Humboldt-Universität zu Berlin, Berlin, Germany; 3 Hochschule Osnabrück, University of Applied Sciences, Osnabrück, Germany

**Keywords:** Health care, Psychology

## Abstract

When exposed to hundreds of medical device alarms per day, intensive care unit (ICU) staff can develop “alarm fatigue” (i.e., desensitisation to alarms). However, no standardised way of quantifying alarm fatigue exists. We aimed to develop a brief questionnaire for measuring alarm fatigue in nurses and physicians. After developing a list of initial items based on a literature review, we conducted 15 cognitive interviews with the target group (13 nurses and two physicians) to ensure that the items are face valid and comprehensible. We then asked 32 experts on alarm fatigue to judge whether the items are suited for measuring alarm fatigue. The resulting 27 items were sent to nurses and physicians from 15 ICUs of a large German hospital. We used exploratory factor analysis to further reduce the number of items and to identify scales. A total of 585 submissions from 707 participants could be analysed (of which 14% were physicians and 64% were nurses). The simple structure of a two-factor model was achieved within three rounds. The final questionnaire (called Charité Alarm Fatigue Questionnaire; CAFQa) consists of nine items along two scales (i.e., the “alarm stress scale” and the “alarm coping scale”). The CAFQa is a brief questionnaire that allows clinical alarm researchers to quantify the alarm fatigue of nurses and physicians. It should not take more than five minutes to administer.

## Introduction

### Background

In intensive care units (ICUs), staff rely on alarms to inform them of potentially dangerous conditions of patients (e.g., high blood pressure), the status of syringe pumps (e.g., medication syringe empty), or technical failures of medical equipment (e.g., battery empty). However, there are so many alarms in ICUs today that staff cannot respond to every alarm^1^. Moreover, few alarms are due to a situation where action is required because artifacts and mismeasurements can also trigger alarms. In a recent survey sent to staff members of all ICUs of a large German university hospital, participants estimated that 56% of all alarms are false or do not require a response^2^, while other studies indicate even higher percentages of 72–99%^3^. Under these circumstances, ICU staff can develop “alarm fatigue,” a condition in which they are desensitised to alarms and respond inadequately or not at all to alarms^4^. Alarm overload is a major cause of stress and dissatisfaction in the ICU workplace and has been shown to pose a significant and life-threatening risk to patients^5^. In the United States alone, hundreds of patient deaths are attributed to alarms not being answered or being answered too late^6^. Accordingly, the Emergency Care Research Institute (ECRI) listed alarm overload among the “Top 10 Health Technology Hazards 2020”^7^.

Alarm fatigue of ICU staff was recognised as a problem more than 20 years ago^8^, yet a gold standard for measuring it has not been established^9,10^. Torabizadeh et al.^11^ made the first and so far the only attempt to systematically construct a reliable instrument for measuring alarm fatigue in nurses. Some studies, of which five were peer-reviewed^12–16^ and two were published as dissertation manuscripts^17,18^, already used Torabizadeh et al.’s questionnaire, while others took it as a resource to build their own custom questionnaire^19^. We welcome this trend towards using a standardised, uniformly agreed-upon instrument in clinical alarm research. In our opinion, a contemporary, standard alarm fatigue questionnaire should fulfil three central conditions, which have only been partially realised in previous approaches. First, it should be transparent about the questionnaire’s validated language; second, it should follow the best practices of scale construction; and third, it should target both nurses and physicians.

#### Condition 1: transparency about the questionnaire’s validated language

We believe that a standardised instrument for measuring alarm fatigue should be explicit about its original language and be meticulously translated before being used in a new language. Low-quality translations often lack the semantic subtleties of the original items or imbue a different meaning altogether^20,21^. For example, Torabizadeh et al. do not specify the original language of their questionnaire and how they arrived at its English translation. Seifert et al. then used Torabizadeh et al.’s English version without questioning the validity of the translation^15^. Alan et al. translated Torabizadeh et al.’s English version into Turkish using a formalised protocol^14^, thereby creating a second-level translation of a non-validated first-level translation. Akturan et al. then used this Turkish version to assess nurses’ alarm fatigue in COVID-19 ICUs^13^. Bourji et al. also followed a formalised protocol in translating the questionnaire into Arabic but did not specify whether the source was the English or the Persian version^12^.

#### Condition 2: following best practices in the scale construction process

Existing studies on alarm fatigue did not adhere to best practices recommended in the literature when designing their questionnaires. They either created their own items (see^11^ for an overview) or used parts of a survey distributed by the Healthcare Technology Foundation (HTF) in 2006^22^ (e.g.^23–27^,). Boateng et al. recommended starting with an item pool that is at least twice as large as the targeted final length of the questionnaire and successively reducing the number of items to the essential ones by means of a theory-driven and statistical approach. This procedure ensures that the questionnaire covers all aspects of the target construct^28^.

#### Condition 3: the target group should be nurses and physicians

We are convinced that both nurses and physicians should be able to voice their opinion since clinical alarm systems are complex and excessive alarms occur due to multiple interacting variables^9^. Developing an alarm fatigue questionnaire only for nurses may create confusion among researchers. For example, even though Torabizadeh et al. developed their questionnaire for nurses, Bourji et al. used it to quantify the alarm fatigue of physicians^12^.

### Aim

With this work, we aim to develop a brief questionnaire that allows clinical alarm researchers to quantify the alarm fatigue of nurses and physicians.

## Methods

### Ethics approval

The ethical approval for this study was granted by the Ethics Commission of the Charité – Universitätsmedizin Berlin (EA4/218/20). This study was performed in accordance with relevant guidelines and regulations. Prior to the study, all participants provided their informed consent.

### Overview

Our methodology is based on the three phases of scale construction of Boateng et al.^28^, where phase one describes the item development, phase two is the scale development, and phase three is the scale evaluation. Due to the convenience of being situated in a large German university hospital, we developed the questionnaire in German. For this article, we translated all items into English using DeepL^29^ and had them proofread by a native English speaker.

The length of the final questionnaire should be approximately 10–15 items to fit on a standard printer page or tablet screen. We chose this target length because the questionnaire should be brief enough so that busy ICU staff can quickly fill it out during their shift – after all, short questionnaires might be completed more often and their questions answered more thoroughly than those of longer questionnaires^30^.

### Item development

We synthesised our definition of alarm fatigue from definitions found in previous literature^4,11^. Hence, we define alarm fatigue as follows:

A sensory overload due to exposure to an excessive number of clinical alarms, which can lead to desensitisation and loss of competence in handling alarm-related procedures (such as dismissing alarms or adjusting monitoring thresholds). Alarm-fatigued ICU staff struggles to identify and prioritise clinical alarms efficiently.

In line with the recommendations by Boateng et al., our aim was to construct an item pool that covers all aspects of that definition and has at least twice as many items as the approximate target length of 10–15. We started by partly developing items ourselves, but mostly derived them from previous studies on alarm fatigue^11,24,25,31–35^. In total, we identified 124 items for our initial item pool. After pruning redundant items and those not directly linked to our definition of alarm fatigue, 35 items remained. All items were translated into German and phrased so that they fit a 5-point Likert response scale with the following options: “I very much agree,” “I agree,” “[I agree] in part,” “I do not agree,” and “I do not agree at all”.

#### Cognitive interviews

In order to evaluate each item’s face validity, relevance to the daily clinical practice, and comprehensibility we conducted cognitive interviews^28,36^. Using convenience sampling, we interviewed 15 representatives from the target group of the questionnaire (13 nurses, two physicians; mean years of ICU experience = 13.9). During the interviews, we went carefully through each item, first asking our interviewees to formulate an answer and then posing at least one of the following questions: What do you think X refers to? (*X* = a certain word or phrase); Why did you answer that way?; How did you come up with your answer?; Are there words that are ambiguous? If yes, how would you rephrase the question? The interviewer took handwritten notes. After all interviews were conducted, we authors met to discuss the remarks for each item. As a result, we merged two redundant items, rephrased or elaborated 23 items with examples, and added five items that covered as yet untouched aspects. Thus, 39 items were submitted to the evaluation by alarm fatigue experts.

#### Expert evaluation

To ensure the “adequacy of content domain sampling”^37^, we asked 32 experts to review the preliminary questionnaire and rate whether the items are relevant and suitable for measuring alarm fatigue in nurses and physicians. We focused on obtaining input from as many experts as possible within a reasonable sample size that allowed for careful qualitative analysis. We used purposive sampling, considering someone an expert on alarm fatigue if they have conducted research on the topic at some point in their career or are experienced in managing alarms in ICUs. The experts were 16 nurses and 14 physicians from our institution. One expert worked in the patient monitoring industry and another one in the aviation industry. All experts were German native speakers. Each expert received a link to an electronic questionnaire (realised in REDCap^38,39^) that asked them to indicate whether an item is “suitable,” “less suitable,” “rather unsuitable,” or “unsuitable” on a 4-point Likert scale. Before commencing, the experts were provided with a brief recapitulation of our definition of alarm fatigue. If they selected the option “(rather) unsuitable,” a text field appeared below the question, providing them with the opportunity to explain their decision. At the end of the survey, experts had the opportunity to mention aspects of alarm fatigue which they felt to be missing or underrepresented by the items.

After receiving the experts’ assessments, we calculated each item’s *P*_*i*_, which is defined as the number of rater-rater pairs in agreement, relative to the number of all possible rater-rater pairs^40^ as well as the proportion of raw agreement. All items rated by at least 75% of the experts as “suitable” and where disagreement was low (defined as a *P*_*i*_ > 0.75) were kept as they were. Three items fell into this category. We decided to remove all items rated by less than 50% of the experts as “suitable.” Two items fell into this category. All other 34 items were discussed by us authors. Based on the experts’ comments, we subsequently rephrased 15 items, deleted 12, and kept seven unchanged. Hence, 27 items were submitted to the scale development phase (see Table 1 in the Supplementary Information for a list of all items). No aspect of alarm fatigue was missing or underrepresented in the items, according to the experts.

### Scale development

In the scale development phase, we collected responses on all 27 items from physicians and nurses using a voluntary response sampling method. Using descriptive statistics and exploratory factor analysis (EFA), we further reduced the number of items and identified scales in the questionnaire.

#### Participant recruitment

Participants who completed the questionnaire were offered to participate in a lottery where they could win a €50 voucher for online shopping. Those who already participated in the cognitive interviews or the expert evaluation of the item development phase were asked not to participate in the survey.

#### Data collection

We pseudo-randomly arranged the 27 items and submitted them as an online survey via REDCap to mailing lists with all staff members of 15 ICUs on the three campuses of the Charité – Universitätsmedizin Berlin between April and June 2021. We sent reminders to fill out the survey approximately every two weeks. All data were collected anonymously, and participants were asked to provide their data processing consent for having their data analysed.

#### Data analysis

We processed and analysed the data in R (Linux version 4.0.3) with the help of Tidyverse^41^, reshape2^42^, psych^43^, and GPArotation^44^. All response options were scored as numbers ranging from − 2 (**≙** “I do not agree at all'') to 2 (≙ “I very much agree”). Items with a negative valence were reverse-scored. In line with Heymans and Eekhout^45^, we imputed data missing at random based on the predictive mean matching algorithm of the mice package^46^ using one imputation. Empty questionnaires and those stopped too early (likely due to survey fatigue) were not imputed because these missing data points do not satisfy the assumption of “missing (completely) at random.” We assumed survey fatigue if a participant did not answer at least the last 15% (i.e., four or more items) of the questionnaire.

##### Descriptive statistics and EFA prerequisites

Before submitting the data to the EFA, we assessed whether it is appropriate using the Kaiser–Meyer–Olkin (KMO) and Bartlett’s test. Items with a limited range (where not all of the answering options were selected), a heavy skew (i.e., a non-normal distribution where the mean ≠ 0), individual KMO values < 0.7 and/or weak correlations with other items (i.e., more than 50% < 0.1) were eliminated. Items with correlations > 0.9 were also eliminated due to the risk of multicollinearity. To spot potential outliers, we calculated each participant’s Mahalanobis distance^47^.

##### Exploratory factor analysis

The aim of our EFA was to reduce the number of items to not more than 15 and to create a “simple structure,” where, after rotation, each factor has at least three loadings |*f*_*ij*_|> 0.3, no item has cross-loadings |*f*_*ij*_|≥ 0.3, and each factor is theoretically meaningful^48^. Therefore, items with loadings |*f*_*ij*_|< 0.3 or cross-loadings |*f*_*ij*_|≥ 0.3 were eliminated. Where objective criteria did not suggest enough items to be eliminated to reach our desired number of items, we assigned similar items into one of seven groups (e.g.: “Avoidable Alarms”; see Table 1 in the Supplementary Information) and only retained those items with the highest loadings per group.

In each iteration of the analysis, we determined the number of factors to extract a priori by considering theoretical plausibility, a visual inspection of the scree plot^49^, parallel analysis^50^, and the Kaiser-Guttman criterion^51^. In line with the suggestions by Flora et al.^52^ for variables measured on an ordinal Likert scale, we estimated our factor analysis model using the unweighted least-squares (ULS) algorithm with polychoric correlations. We rotated the factor loadings using an oblique method (i.e., direct oblimin) since our theory suggests that the resulting factors correlate.

### Scale evaluation

As a measure of internal homogeneity, we report Cronbach’s coefficient alpha. To underpin the construct validity of the scale, we attempted to measure convergent validity by asking participants to rate their own alarm fatigue between 0% (not alarm fatigued at all) and 100% (extremely alarm fatigued) at the end of the survey. We also provided a brief recapitulation of our definition of alarm fatigue below the question to help participants in their self-assessment. We correlated the results of each participant’s self-reported alarm fatigue with their mean score of the final (post-EFA) questionnaire items, assuming that a high correlation indicates convergent validity.

## Results

**Figure 1:**
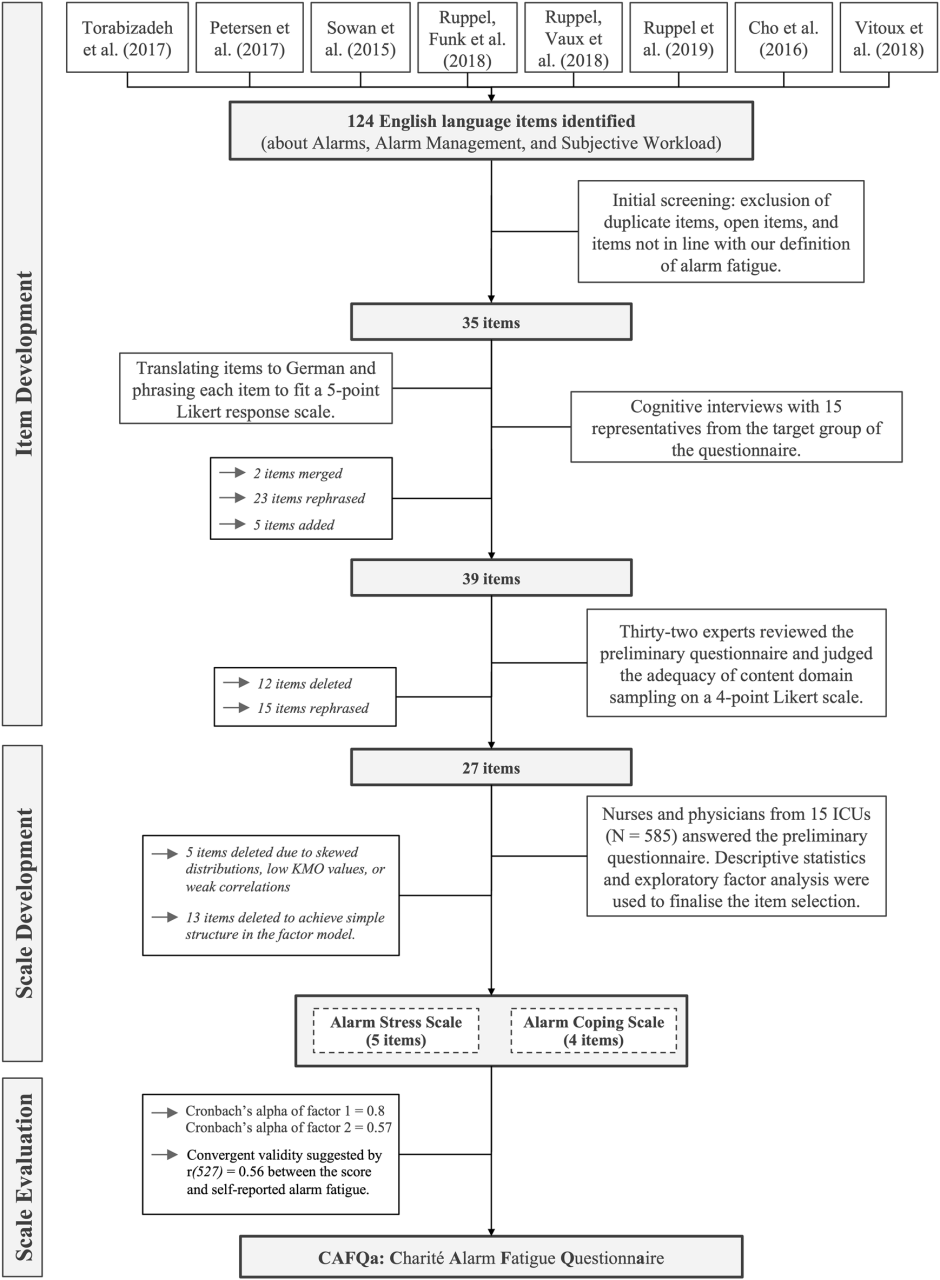
An overview of our procedure and the results divided into the phases of scale construction by Boateng et al.^28^.

In total, 707 healthcare professionals participated in the survey, of which 78 questionnaires were handed in empty. Forty-four participants showed survey fatigue. Forty participants had at least one item response missing at random (0.41% missing data in total). In total, we included 585 submissions in our analysis. Figure 1 shows an overview of our results at each stage of the questionnaire construction process.

### Scale development

#### Descriptive statistics and EFA prerequisites

Mardia’s test rejected the null hypothesis that the multivariate skew and kurtosis are drawn from a normal distribution with both *p* < 0.001. Because we could not assume multivariate normality and because our items were based on an ordinal Likert scale, we relied on polychoric correlations as the basis of our factor analysis^48,52^. To spot potential outliers, we calculated each subject’s Mahalanobis distance. Ten outliers were detected (χ^2^(27) cutoff = 55.48, *p* < 0.001). However, a visual inspection of each of the 10 subjects’ scores did not reveal an abnormal response pattern and our sample size is large enough to mitigate potential influences. Thus, we did not remove any outliers from the data. The KMO statistic ^53^ is 0.86 and thus above the recommended value of 0.8. Three items (10, 19, and 27) showed low KMO values (< 0.7). Bartlett’s test of sphericity^48^ rejected the null hypothesis that the correlation matrix is an identity matrix (χ^2^(351) = 5710.51, *p* < 0.001). Multicollinearity did not seem to be present, as the determinant of the R matrix was greater than 0.00001^54^ and no correlation was greater than |.7|. These results suggested that the data is adequate for factor analysis.

##### Item elimination based on descriptive statistics

We removed items 1 and 10 due to their skewed distribution and items 19 and 27 due to their low KMO values (< 0.7). We further removed item 22 because it had overall few and generally weak correlations with other items.

##### Determining the initial number of factors to extract

Parallel analysis suggested six factors to extract, while a visual inspection of the scree plot suggested between one and three. The Kaiser-Guttman criterion suggested 3 factors to extract. Our definition of alarm fatigue suggested three dimensions (items related to an excessive number of alarms, items related to incompetence in handling alarms and monitors, and items related to desensitisation). Therefore, we decided to first compare a three-factor model with a two-factor one.

#### Results of the EFA

The desired questionnaire length of not more than 10–15 items was achieved in three rounds.

##### Factor analysis round one

Starting with a three-factor model, all items related to interruptions by alarms (13, 8, and 25) constructed their own factor. Due to their similarity and because our definition of alarm fatigue does not suggest a scale dedicated to measuring interruptions by alarms (we would expect these items to be related to the scale regarding an excessive number of alarms), we proceeded with a two-factor solution. The two-factor solution almost achieved a simple structure as defined above. Only the loading of item 9 was slightly below |0.3|. Since we aimed at reducing the questionnaire to a length of 10–15 items and no objective criteria allowed us to eliminate the required number of items, we decided to group items with similar content and only retain those with the highest loading(s) per group (Table 1 in the Supplementary Information shows which item was assigned to which group).

In the group “Avoidable Alarms,” item 23 had the higher loading; therefore, item 16 was eliminated. In the group “Being able to care for patients,” items 4 and 25 both loaded strongly on factor 1, as did items 13 and 8. However, the latter two were eliminated due to their similarity to item 25. In the group “Bad Coping,” item 21 had the higher loading; therefore, item 17 was eliminated. Items 3 and 18 from the group “Good Coping” are very similar, while 6 collects new information. Item 3 had the lower loading on factor 2 and was therefore eliminated. In the group “Pinpoint source,” all items except 12 had relatively weak loadings; therefore, 9, 14 and 24 were eliminated. In the group “Psychological symptoms,” item 15 loads strongly on factor 1, as do 5 and 26. All three items capture slightly different aspects and were therefore not eliminated. Items 20 and 2 did not load as highly and as uniquely on factor 1 and were therefore eliminated.

##### Factor analysis round two

After eliminating ten items in round one, parallel analysis suggested three factors to extract, while a visual inspection of the scree plot suggested one or two. The Kaiser-Guttman criterion suggested two factors as well. We therefore decided on a two-factor model, which achieved a simple structure as defined above. However, we removed item 7 due to its comparatively weak loading on factor 1 and a simultaneous cross-loading close to 0.3 on factor 2. We also decided to delete item 23, due to its light cross-loading on factor 2 and because we consider its content superfluous (false alarms are normal and very common in ICUs, which is also suggested by its skewed distribution). Finally, we decided to remove item 21, because of its low loading on factor 1 and low communality, and because in our opinion it is not essential for measuring alarm fatigue from a theoretical point of view.

##### Factor analysis round three

Having eliminated an additional three items in round two, parallel analysis and the Kaiser-Guttman criterion suggested two factors to extract, while a visual inspection of the scree plot suggested one or two. We decided on a two-factor model again, also with regard to our results above, which finally achieved a simple structure (Table 1). In the rotated model, factor 1 accounted for 28.25% of the total variance and 66.96% of the common variance while factor 2 accounted for 13.94% of the total variance and 33.04% of the common variance. Both factors were correlated with r = 0.27, which justifies having chosen an oblique rotation.

**Table 1:** Pattern coefficients of the final two-factor model. Notice that all reverse-scored items load on factor 2.

Item no	Item	Factor 1	Factor 2	Communality
4	With too many alarms on my ward, my work performance and motivation decreases	**0.698**	0.055	0.52
5	Too many alarms trigger physical symptoms for me, e.g., nervousness, headaches, sleep disturbances	**0.745**	− 0.130	0.50
6	In my ward, a procedural instruction on how to deal with alarms is regularly updated and shared with all staff^a^	− 0.040	**0.449**	0.19
11	Responsible personnel respond quickly and appropriately to alarms^a^	0.031	**0.708**	0.52
12	The audible and visual monitor alarms used on my ward floor and cockpit allow me to clearly assign patient, unit, and urgency^a^	0.011	**0.441**	0.20
15	Alarms reduce my concentration and attention	**0.835**	0.063	0.74
18	Alarm limits are regularly adjusted based on patients' clinical symptoms (e.g., blood pressure limits for condition after bypass surgery)^a^	− 0.006	**0.562**	0.31
25	My or neighbouring patients' alarms or crisis alarms frequently interrupt my workflow	**0.570**	0.087	0.37
26	There are situations when alarms confuse me	**0.682**	− 0.035	0.45

^a^Item with a negative valence that is reversely scored.

The bold values represent the factor loadings of the items on their respective factors.

### Scale evaluation

Cronbach’s alpha of factor 1 was 0.8 (95% CI = 0.78–0.83) and 0.57 (95% CI = 0.51–0.62) for factor 2. Cronbach’s alpha across factors was 0.74 (95% CI = 0.71–0.77). Alpha would not increase for either factor or across factors if any item was deleted.

The participants’ mean scores correlated strongly with the self-reported alarm fatigue in percent: *r*(527) = 0.56 (*p* < 0.001; 95% CI = 0.5–0.62). As did the scores of factor 1: *r*(527) = 0.54, (*p* < 0.001; 95% CI = 0.48–0.6). The scores of factor 2 correlated with the self-reported alarm fatigue less than the scores of factor 1: *r*(527) = 0.3, (*p* < 0.001; 95% CI = 0.22–0.37). Of 585 participants, 56 did not provide their self-reported alarm fatigue.

## Discussion

Clinical alarm researchers lack a gold standard for measuring alarm fatigue in nurses and physicians. Hence, our aim was to design and evaluate a brief questionnaire to fill this gap. We achieved this by submitting an initial item pool of 124 items to a rigorous selection process. Cognitive interviews with the target group ensured the face validity of all items. Thirty-nine experts ensured that the items adequately represent the content domain of alarm fatigue. We collected questionnaire responses from 707 participants, of which 585 could be submitted to an EFA. A simple structure was achieved in three iterations. The final questionnaire consists of nine items along two factors with Cronbach’s alpha = 0.8 and 0.57 for factor 1 and factor 2, respectively. The convergent validity of the questionnaire is suggested by a strong correlation between the participants’ score on the questionnaire and their self-reported alarm fatigue. We named the questionnaire “Charité Alarm Fatigue Questionnaire” (abbreviated to CAFQa, pronounced like the author Franz Kafka). With the Likert options being coded from zero to four (zero being “I do not agree at all” and four being “I very much agree”), the score ranges from 0 (no alarm fatigue at all) to 36 (extreme alarm fatigue), with 18 being the midpoint. We suggest expressing the alarm fatigue as a percentage, as this is more intuitive. We provide a print-ready version of the questionnaire in the Supplementary Information.

### Interpretation of the findings

Factor 1 is described by items concerning the psychophysiological effects of excessive alarms (i.e., reduced motivation and concentration, physical malaise, and confusion). Factor 2 is described by items covering structural and systemic aspects that contribute to excessive alarms (i.e., responsibilities, procedures, and the monitoring setup). Definitions in the literature so far have focused mainly on the psychophysiological aspects of alarm fatigue (e.g.^4^). However, the questionnaires mentioned above (Introduction, paragraph 2) also included items about alarm management procedures. Our results highlight that indeed both aspects should be captured to quantify alarm fatigue. For example, some individuals might not notice or might misattribute the effects of excessive alarms on their work performance (likely resulting in a low score on factor 1), while simultaneously having a low score on factor 2. This pattern would suggest that alarm fatigue is present after all. We recommend referring to items on factor 1 as being on the “alarm stress scale” and to items on factor 2 as being on the “alarm coping scale”.

### Recommendations for future research

Future research should test whether our hypothesised dimensionality can be replicated (e.g., by means of confirmatory factor analysis)^28^. In Table 1, the communalities of most items on factor 2 are rather low, suggesting that they do not add as much information as the items on factor 1. Thus, confirmatory factor analysis on a new sample might lead to reducing the questionnaire to one scale only. Replicating the two scales successfully, however, would indicate the CAFQa’s content validity and raise further interesting questions, such as the following: To what extent can the two aspects of alarm fatigue be investigated independently? Would it be possible to dissociate them? For example, one ICU might score high on the alarm coping scale (i.e., it has well-maintained procedures for managing alarms) while also scoring high on the alarm stress scale (i.e., staff still feels negatively impacted by alarms). This is what happened in the study by Sowan et al.^55^. The authors re-educated bedside nurses on monitor use and changed the default alarm settings of the cardiac monitors, thereby effectively reducing the total number of alarms. However, the staff's attitude towards alarms did not improve. We are optimistic that our questionnaire would be able to unearth this dissociation.

Important next steps for establishing CAFQa as the gold standard for measuring alarm fatigue are finding out whether additional validities can be established (e.g., discriminant validity or criterion validity) and demonstrating test–retest reliability. We also recommend developing translations by following a standardised procedure (e.g., as proposed by Harkness et al.^20^). We already phrased the items neutrally to make them applicable across the globe regardless of culture-specific ICU regulations (e.g., by omitting the subject in item 18, or by speaking of “responsible personnel” in item 11). However, translators should take care that each item fits the target culture. In the ICUs of our study setting, physicians are (besides nurses) heavily involved in alarm management. Hence, we deliberately developed the questionnaire to measure the alarm fatigue for nurses and physicians, even though in some cultures only nurses deal with alarms. In that case, physicians cannot be alarm fatigued. The CAFQa can account for that because physicians would then respond in a way that they receive a low alarm fatigue score (e.g., by disagreeing with items on the alarm stress scale and by agreeing with items on the alarm coping scale). We are curious to see whether future cross-cultural research can confirm this hypothesis.

### Limitations

Our initial pool of items might be biased because we did not review the literature within a formal framework (e.g., a systematic or scoping review). However, we aimed to reduce that bias by making sure that all aspects of our a priori definition of alarm fatigue are covered in the item pool and by submitting the item pool to a rigorous selection process. Future research should conduct a systematic review to find out whether the CAFQa should be expanded to cover yet untouched aspects of alarm fatigue.

We might have introduced research bias because we relied on non-probability sampling techniques for the cognitive interviews and expert evaluation. Additionally, only two physicians participated in the interviews, tipping the balance in the sample strongly toward nurses. However, the interviews were only one part of a series of repeated evaluations. The physicians' perspective was also covered during the expert evaluations and by the physicians among us authors throughout the item development phase. Item design is not a quantitative science where large samples are needed for inferences about a population. Instead, we prioritised taking the time for careful analyses of the interview notes and expert comments.

Our voluntary response sampling method for the survey has potentially introduced a self-selection bias: ICU staff who are more affected by alarms might have been more likely to participate. They might also have exaggerated their responses to items, expecting that this signals a need for change in their ICU.

At first glance, it might appear as if the two-factor solution of the final model solely emerged due to the scoring direction of the items, since all reverse-scored items load on factor 2. However, we believe this is partly due to chance and partly due to the fact that all items related to systemic aspects of alarm fatigue were phrased in a nonnegative manner, to prevent misunderstandings^56–58^. The positive correlation (due to the reverse scoring) between factor 2 and participants’ self-reported alarm fatigue suggests that factor 2 is indeed related to alarm fatigue and not an artifact of the items’ scoring direction. Although our assumption that participants can accurately reflect on their own alarm fatigue might be flawed, we do have the impression that ICU nurses and physicians have heard of the concept of alarm fatigue and can readily report how excessive alarms make them feel.

## Conclusion

We developed a questionnaire for measuring alarm fatigue in nurses and physicians that consists of nine items answered on a single 5-point Likert response scale and should take not more than five minutes to administer. Our results suggest that alarm fatigue should be measured on two distinct scales, covering the psychophysiological effects of alarms as well as staff’s coping strategies. We hope our work proves itself useful to clinical alarm researchers and finally allows for a standardised approach to quantifying the alarm burden experienced by ICU staff.

### Supplementary Information

Supplementary Information 1.

Supplementary Information 2.

Supplementary Information 3.

## Data Availability

The datasets generated during and analysed during the current study are available from the corresponding author upon reasonable request.

## Abbreviations

CAFQa: Charité Alarm Fatigue Questionnaire
CI: Confidence interval
EFA: Exploratory factor analysis
e.g.: For example
ICU: Intensive care unit
i.e.: That is
KMO: Kaiser–Meyer–Olkin
