# Supplementary material for: A brief questionnaire for measuring alarm fatigue in nurses and physicians in intensive care units
Source: Sci Rep. 2023 Aug 24;13:13860. doi: 10.1038/s41598-023-40290-7 (PMC10449802; doi:10.1038/s41598-023-40290-7)
Supplement: Supplementary file 1 — Supplementary Information 1. [file 41598_2023_40290_MOESM1_ESM.pdf]

# Supplementary Information

# A Brief Questionnaire for Measuring Alarm Fatigue in Nurses and Physicians in Intensive Care Units

Maximilian Markus Wunderlich[a,1], Sandro Amende-Wolf [a,1,2], Henning Krampe[2], Jochen Kruppa[3], Claudia Spies [2], Björn Weiß [2], Belinda Memmert[2], Felix Balzer[1,a], Akira-Sebastian Poncette[1,2,a, *]

[1] *Institute for Medical Informatics, Charité – Universitätsmedizin Berlin, Corporate Member of Freie Universität Berlin and Humboldt-Universität zu Berlin, Berlin, Germany*

[2] *Department of Anesthesiology and Intensive Care Medicine, Charité – Universitätsmedizin Berlin, Corporate Member of Freie Universität Berlin, Humboldt-Universität zu Berlin, Berlin, Germany*

*3 Hochschule Osnabrück, University of Applied Sciences, Osnabrück, Germany*

[a]These authors contributed equally to this work

*corresponding author

E-Mail: akira-sebastian.poncette@charite.de

## Participant Demographics

In total, we included 585 submissions in our analysis. Among these, 486 (83%) indicated to work more than eight days per month in an ICU, 87 (15%) indicated to work not more than six to eight days per month in an ICU. Twelve (2%) did not state their monthly time on ICU. Eighty-three (14%) of the participants were physicians, 375 (64%) were nurses, 112 (19%) were assistant nurses, nurses in training, medical students, or interns. Fifteen (3%) refused to provide their profession. Most participants (410; 70%) stated to have one or more year(s) of ICU experience, 127 (22%) were ICU experienced for up to one year, 35 (6%) for up to two months. Thirteen (2%) did not provide their ICU experience. The figure below visualises the participants' demographics.

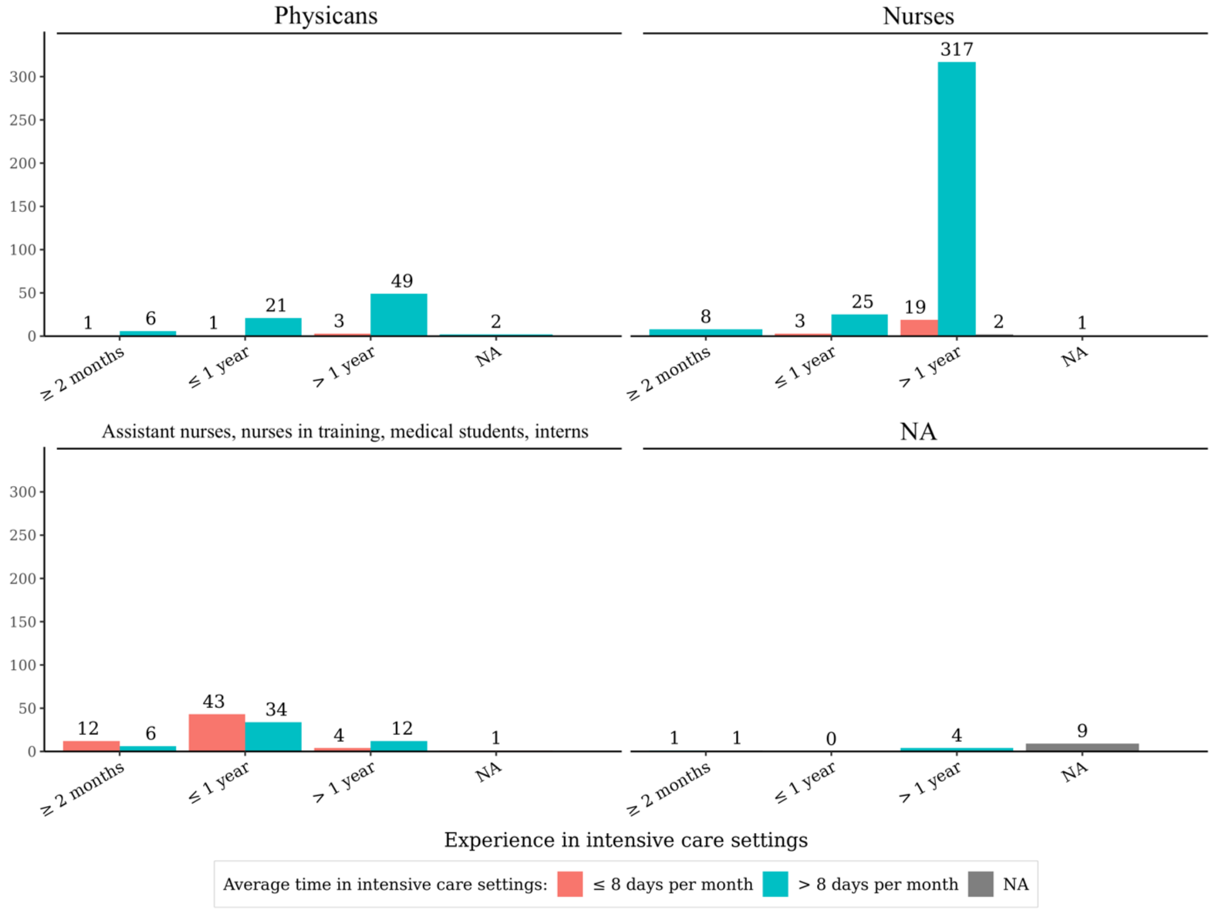


**Supplementary Figure 1**. Profession, ICU experience and average monthly ICU time of the 585 participants whose questionnaires were submitted to the analysis. Most participants were experienced nurses who spent more than one week per month in an ICU.

**Supplementary Table 1.** Overview of the 27 items that we submitted to the scale development phase.

| No. | Item | Item Group | Factor |
|---|---|---|---|
| 1 | Alarms are too frequent in my ward. | Too many alarms | *NA* |
| 2 | Frequent false readings that trigger alarms slow down my reaction time. | Psych. symptoms | *NA* |
| 3 | The alarm limits are individually adapted to the patient's clinical condition.[a] | Good coping | *NA* |
| 4 | With too many alarms on my ward, my work performance and motivation decreases. | Being able to care for patients | **1** |
| 5 | Too many alarms trigger physical symptoms for me, e.g., nervousness, headaches, sleep disturbances. | Psych. symptoms | **1** |
| 6 | In my ward, a procedural instruction on how to deal with alarms is regularly updated and shared with all staff.[a] | Good coping | **2** |
| 7 | Clinical crisis alarms ('red alarms') are too common on my ward. | Too many alarms | *NA* |
| 8 | Alarms prevent me from being able to perform other tasks (administering medications, nursing care, etc.). | Being able to care for patients | *NA* |
| 9 | It is difficult to identify which medical device at the patient's bedside is triggering a relevant alarm. | Pinpoint source | *NA* |
| 10 | I check the alarm limits at the beginning of the shift.[a] | Good coping | *NA* |
| 11 | Responsible personnel respond quickly and appropriately to alarms.[a] | *NA*[b] | **2** |
| 12 | The audible and visual monitor alarms used on my ward floor and cockpit allow me to clearly assign patient, unit and urgency.[a] | Pinpoint source | **2** |
| 13 | Alarms or crisis alerts frequently interrupt my care of my patients. | Being able to care for patients | *NA* |
| 14 | I feel like I often don't notice alarms from the syringe pumps and have a delayed reaction to them. | Pinpoint source | *NA* |
| 15 | Alarms reduce my concentration and attention. | Psych. symptoms | **1** |
| 16 | Activities close to the patient (e.g., blood sampling, mobilization, aspiration of tracheal secretions) result in an unnecessary number of alarms. | Avoidable alarms | *NA* |
| 17 | I respond immediately only to clinical crisis alarms ("red alarms"). | Bad coping | *NA* |
| 18 | Alarm limits are regularly adjusted based on patients' clinical symptoms (e.g., blood pressure limits for condition after bypass surgery).[a] | Good coping | **2** |
| 19 | On my ward, only my professional group responds to alarms. | *NA*[b] | *NA* |

| | | | |
|---|---|---|---|
| 20 | When alarms go off repeatedly, I become indifferent to them. | Psych. symptoms | *NA* |
| 21 | To avoid a flood of alarms, I adjust the alarm limits generously. | Bad coping | *NA* |
| 22 | If alarms occur too frequently, I discuss further action with colleagues.[a] | Good coping | *NA* |
| 23 | Alarms are often triggered even when there is no risk to patients. | Avoidable alarms | *NA* |
| 24 | The physical layout (including size) of my ward affects my perception and response to syringe pump or monitor alarms. | Pinpoint source | *NA* |
| 25 | My or neighbouring patients' alarms or crisis alarms frequently interrupt my workflow. | Being able to care for patients | **1** |
| 26 | There are situations when alarms confuse me. | Psych. symptoms | **1** |
| 27 | On my ward, dealing with alarms and alarm settings (alarm management) is a regular topic.[a] | Good coping | *NA* |

[a] Item with a negative valence that was reversely scored.

[b] Item that did not fit into any of the defined groups and was treated independently.

**Supplementary Table 2.** Descriptive statistics for each item.

| Item | Mean | Median | SD | Kurtosis | Skew |
|---|---|---|---|---|---|
| 1 | 1.25 | 1 | 0.87 | -0.15 | -0.87 |
| 2 | 0.76 | 1 | 0.94 | -0.27 | -0.46 |
| 3 | -0.78 | -1 | 0.93 | -0.37 | 0.41 |
| 4 | 0.14 | 0 | 1.10 | -0.86 | 0.09 |
| 5 | 0.26 | 0 | 1.22 | -0.98 | -0.18 |
| 6 | 0.72 | 1 | 0.99 | -0.45 | -0.51 |
| 7 | 0.64 | 1 | 01.02 | -0.64 | -0.38 |
| 8 | 0.64 | 1 | 0.99 | -0.41 | -0.43 |
| 9 | -0.50 | -1 | 01.02 | -0.06 | 0.60 |
| 10 | -0.95 | -1 | 1.17 | -0.05 | 0.95 |
| 11 | -0.21 | 0 | 0.88 | 0.16 | -0.01 |
| 12 | -0.23 | 0 | 01.02 | -0.49 | 0.23 |
| 13 | 0.80 | 1 | 0.87 | -0.34 | -0.39 |
| 14 | -0.41 | -1 | 01.04 | -0.57 | 0.46 |
| 15 | 0.41 | 0 | 01.06 | -0.72 | -0.20 |
| 16 | 0.70 | 1 | 01.02 | -0.46 | -0.49 |
| 17 | 0.05 | 0 | 01.09 | -0.98 | -0.05 |
| 18 | -0.56 | -1 | 0.92 | -0.19 | 0.32 |
| 19 | 0.43 | 1 | 1.25 | -1.04 | -0.34 |
| 20 | 0.21 | 0 | 0.95 | -0.33 | -0.12 |
| 21 | -0.36 | 0 | 0.93 | -0.21 | 0.36 |
| 22 | -0.35 | -1 | 01.05 | -0.42 | 0.43 |
| 23 | 01.01 | 1 | 0.82 | -0.25 | -0.48 |
| 24 | 0.71 | 1 | 01.09 | -0.43 | -0.62 |
| 25 | 0.71 | 1 | 0.91 | -0.20 | -0.43 |
| 26 | 0.16 | 0 | 01.08 | -0.88 | -0.04 |
| 27 | 0.45 | 1 | 1.00 | -0.60 | -0.30 |

# CAFQa: Charité Alarm Fatigue Questionnaire

| No. | Item | I do not agree at all | I do not agree | I agree in part | I agree | I very much agree |
|---|---|---|---|---|---|---|
| 1. | With too many alarms on my ward, my work performance and motivation decreases. | ☐ | ☐ | ☐ | ☐ | ☐ |
| 2. | Too many alarms trigger physical symptoms for me, e.g., nervousness, headaches, sleep disturbances. | ☐ | ☐ | ☐ | ☐ | ☐ |
| 3. | Alarms reduce my concentration and attention. | ☐ | ☐ | ☐ | ☐ | ☐ |
| 4. | My or neighbouring patients' alarms or crisis alarms frequently interrupt my workflow. | ☐ | ☐ | ☐ | ☐ | ☐ |
| 5. | There are situations when alarms confuse me. | ☐ | ☐ | ☐ | ☐ | ☐ |
| 6. | In my ward, a procedural instruction on how to deal with alarms is regularly updated and shared with all staff. * | ☐ | ☐ | ☐ | ☐ | ☐ |
| 7. | Responsible personnel respond quickly and appropriately to alarms.* | ☐ | ☐ | ☐ | ☐ | ☐ |
| 8. | The audible and visual monitor alarms used on my ward floor and cockpit allow me to clearly assign patient, unit, and urgency.* | ☐ | ☐ | ☐ | ☐ | ☐ |
| 9. | Alarm limits are regularly adjusted based on patients' clinical symptoms (e.g., blood pressure limits for condition after bypass surgery).* | ☐ | ☐ | ☐ | ☐ | ☐ |

All items are scored as numbers ranging from 0 (≘ "*I do not agree at all*") to 4 (≘ "*I very much agree*"). Items marked with a * have a negative valence and are scored in the opposite manner.

Alarm Fatigue Scale

0 — 18 — 36

Min. Alarm Fatigue Score — Midpoint — Max. Alarm Fatigue Score

If you have questions, feel free to contact us at data-science@charite.de. Please make sure to cite this article appropriately if you are using this questionnaire.

# CAFQa: Charité Alarm Fatigue Questionnaire

| | | Ich stimme gar nicht zu | Ich stimme nicht zu | Teils/Teils | Ich stimme zu | Ich stimme sehr zu |
|---|---|---|---|---|---|---|
| 1. | Durch zu viele Alarme auf meiner Station nimmt meine Arbeitsleistung und Motivation ab. | ☐ | ☐ | ☐ | ☐ | ☐ |
| 2. | Zu viele Alarme lösen bei mir körperliche Symptome aus, z.B. Nervösität, Kopfschmerzen, Schlafstörungen. | ☐ | ☐ | ☐ | ☐ | ☐ |
| 3. | Alarme reduzieren meine Konzentration und Aufmerksamkeit. | ☐ | ☐ | ☐ | ☐ | ☐ |
| 4. | Die Alarme oder Krisenalarme meiner oder benachbarter Patient:innen unterbrechen häufig meine Arbeitsabläufe. | ☐ | ☐ | ☐ | ☐ | ☐ |
| 5. | Es gibt Situationen, in denen mich Alarme durcheinanderbringen. | ☐ | ☐ | ☐ | ☐ | ☐ |
| 6. | Auf meiner Station wird eine Verfahrensanweisung zum Umgang mit Alarmen regelmäßig aktualisiert und mit allen Mitarbeiter:innen geteilt.* | ☐ | ☐ | ☐ | ☐ | ☐ |
| 7. | Das zuständige Personal reagiert schnell und angemessen auf Alarme.* | ☐ | ☐ | ☐ | ☐ | ☐ |
| 8. | Die auf meinem Stationsflur und meiner Stationskanzel verwendeten akustischen und optischen Monitoralarme erlauben mir die eindeutige Zuordnung zu Patient*in, Gerät und Dringlichkeit.* | ☐ | ☐ | ☐ | ☐ | ☐ |
| 9. | Die Alarmgrenzen werden regelmäßig auf Grundlage der klinischen Symptome der Patient*innen (z.B. Blutdruckgrenzen bei Zustand nach Bypass-Operation) angepasst.* | ☐ | ☐ | ☐ | ☐ | ☐ |

Alle Items werden von 0 (≘ "*Ich stimme überhaupt nicht zu*") bis 4 (≘ "*Ich stimme sehr zu*") bewertet. Die mit einem * gekennzeichneten Items haben eine negative Valenz und werden in umgekehrter Weise bewertet.

Alarm Fatigue Scale

| 0 | 18 | 36 |
|---|---|---|
| Min. Alarm Fatigue Score | Midpoint | Max. Alarm Fatigue Score |

Wenn Sie Fragen haben, können Sie uns gerne kontaktieren: data-science@charite.de. Bitte stellen Sie sicher, dass Sie diesen Artikel angemessen zitieren, wenn Sie diesen Fragebogen verwenden.
